# The pluripotency factor LIN28 marks undifferentiated spermatogonia in mouse

**DOI:** 10.1186/1471-213X-9-38

**Published:** 2009-06-29

**Authors:** Ke Zheng, Xin Wu, Klaus H Kaestner, Peijing Jeremy Wang

**Affiliations:** 1 Department of Animal Biology, University of Pennsylvania School of Veterinary Medicine, Philadelphia, PA 19104, USA; 2 Department of Genetics, University of Pennsylvania School of Medicine, Philadelphia, PA 19104, USA

## Abstract

**Background:**

Life-long production of spermatozoa depends on spermatogonial stem cells. Spermatogonial stem cells exist among the most primitive population of germ cells – undifferentiated spermatogonia. Transplantation experiments have demonstrated the functional heterogeneity of undifferentiated spermatogonia. Although the undifferentiated spermatogonia can be topographically divided into A_s _(single), A_pr _(paired), and A_al _(aligned) spermatogonia, subdivision of this primitive cell population using cytological markers would greatly facilitate characterization of their functions.

**Results:**

In the present study, we show that LIN28, a pluripotency factor, is specifically expressed in undifferentiated spermatogonia (A_s_, A_pr_, and A_al_) in mouse. *Ngn3 *also specifically labels undifferentiated spermatogonia. We used *Ngn3*-GFP knockin mice, in which GFP expression is under the control of all *Ngn3 *transcription regulatory elements. Remarkably, *Ngn3*-GFP is only expressed in ~40% of LIN28-positive A_s _(single) cells. The percentage of *Ngn3*-GFP-positive clusters increases dramatically with the chain length of interconnected spermatogonia.

**Conclusion:**

Our study demonstrates that LIN28 specifically marks undifferentiated spermatogonia in mice. These data, together with previous studies, suggest that the LIN28-expressing undifferentiated spermatogonia exist as two subpopulations: *Ngn3*-GFP-negative (high stem cell potential) and *Ngn3*-GFP-positive (high differentiation commitment). Furthermore, *Ngn3*-GFP-negative cells are found in chains of *Ngn3*-GFP-positive spermatogonia, suggesting that cells in the A_al _spermatogonia could revert to a more primitive state.

## Background

Spermatogenesis is a productive self-renewing system of adult stem cells that continuously generates spermatozoa through life. At the foundation of this system is the spermatogonial stem cells (SSCs) [1-4]. In mouse testis, isolated A (single) spermatogonia (A_s_) are believed to be the most primitive cells and contain the stem cells. In normal situations, while half of A_s _cells divide and give rise to A_pr _(paired) spermatogonia that are interconnected by cytoplasmic bridges due to incomplete cytokinesis, the remaining half of A_s _cells undergo self-renewal divisions. The A_pr _spermatogonia further divide to become chains of 4, 8, 16, or 32 A_al _(aligned) spermatogonia. The A_s_, A_pr_, and A_al _spermatogonia can only be identified by their topographical configurations on the basement membrane of the seminiferous tubules and are collectively referred to as "undifferentiated" spermatogonia, although this nomenclature causes confusion because this population contain both progenitor cells that undergo differentiation and stem cells that are truly undifferentiated [5]. The A_al _spermatogonia differentiate into A1 spermatogonia, which undergo six cell divisions before entering meiosis via A2, A3, A4, Intermediate, and B spermatogonia. The transition from A_al _(undifferentiated) to A1 (differentiating) is a sensitive step during spermatogonial development, as it can be disrupted by several conditions such as cryptorchidism and Vitamin A deficiency [3]. Spermatogonial transplantation along with other studies have demonstrated that SSCs are a subpopulation of undifferentiated spermatogonia, most likely A_s _cells, but not differentiating spermatogonia (A1 to B) [3,6]. Subdivision of the undifferentiated spermatogonia using cytological markers would greatly facilitate characterization of this unique cell population, but so far has not been achieved.

We previously identified *Lin28 *(formerly called *Tex17*) as a gene differentially expressed in mouse spermatogonia by a cDNA subtraction screen [7]. *Lin28 *is predominantly expressed in primitive type A spermatogonia [8]. *Lin28*, encoding an evolutionarily conserved small RNA-binding protein, was first identified as a key regulator of developmental timing in *C. elegans *[9,10]. In *C. elegans*, *Lin28 *is expressed in early larval stage but is rapidly suppressed during embryogenesis and in adult animals by the lin-4 microRNA and the Lin-14 protein [11]. Recently, LIN28 was used together with OCT4, SOX2, and NANOG to reprogram human somatic cells into pluripotent stem cells [12]. In mice, *Lin28 *is expressed in diverse embryonic tissues, embryonic stem cells, and embryonic carcinoma cells, but not in most adult tissues [10,13]. Collectively, these studies have demonstrated that the expression of *Lin28 *is associated with pluripotency.

In this report, we find that *Lin28 *is specifically expressed in the undifferentiated spermatogonia (A_s _to A_al_) of adult mouse testis. Our analysis of *Lin28 *and *Ngn3 *suggests that *Lin28*-expressing undifferentiated spermatogonia can be cytologically divided into two subpopulations: *Ngn3*-GFP-negative spermatogonia that contain high stem cell activity/potential and *Ngn3*-GFP-positive cells that are more committed to differentiation.

## Results

### *Lin28 *is specifically expressed in germ cells in the testis

**Figure 1:**
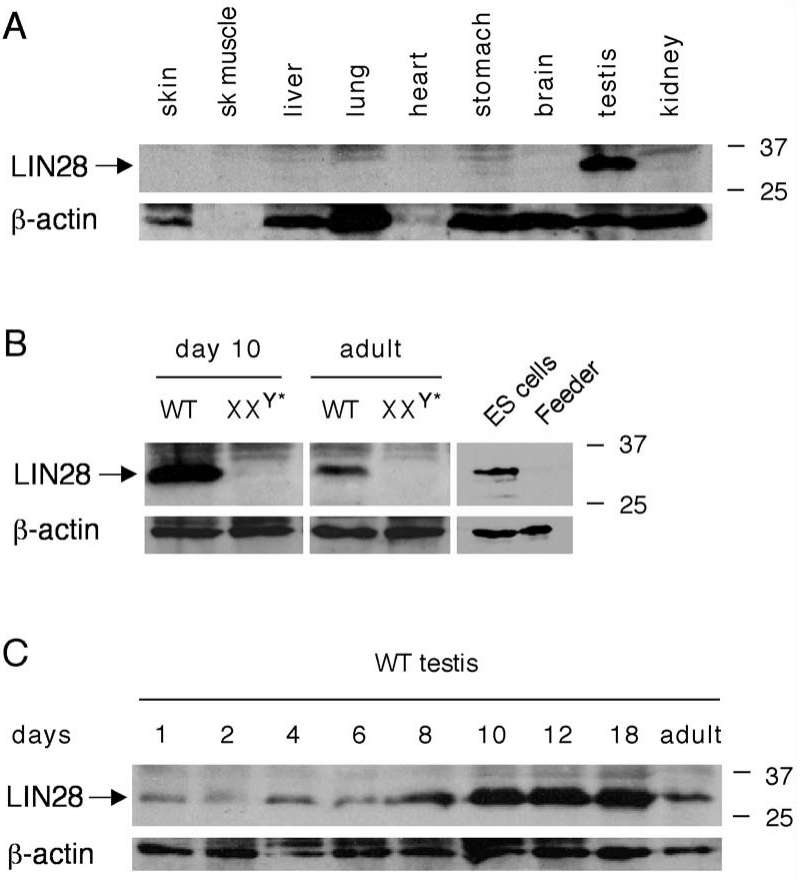
**Expression of LIN28 in mouse testis**. Western blot analysis was performed on 20 μg of protein extracts for each sample. β-actin served as a control. Molecular weight standards were marked in kDa. (**A**) Western blot analysis of LIN28 in adult mouse tissues. (**B**) Absence of LIN28 in germ cell-deficient XX^Y^* testes. Testes were collected from adult and post-natal day 10-old mice. V6.5 mouse embryonic stem (ES) cells served as a positive control. LIN28 was absent in fibroblast feeder cells. (**C**) Developmental expression of LIN28 in postnatal testes. Testes were collected from mice of postnatal day 1 through adulthood.

We cloned *Lin28 *(previously known as *Tex17*) from mouse spermatogonia in a cDNA subtraction screen [7]. Semi-quantitative RT-PCR analysis using enriched germ cell populations showed that the expression of *Lin28 *in testis is restricted to spermatogonia [8]. Western blot analysis of a panel of adult mouse tissues revealed that the LIN28 protein is abundantly expressed in testis but not in other tissues examined (Fig. 1A). The testis of XX^Y* ^male mice completely lack germ cells but contain somatic cells such as Sertoli and Leydig cells [14]. The absence of LIN28 in XX^Y^* testes demonstrated that LIN28 was germ cell-specific (Fig. 1B), in agreement with previous studies [7]. As controls, LIN28 protein was abundant in mouse embryonic stem cells but absent in fibroblast feeder cells (Fig. 1B). We examined the relative protein level of LIN28 in juvenile testes. LIN28 was detectable in testes right after birth, increased its abundance with age, and was most abundant around puberty (day 12 – 18) (Fig. 1C).

### LIN28 marks undifferentiated spermatogonia

**Figure 2:**
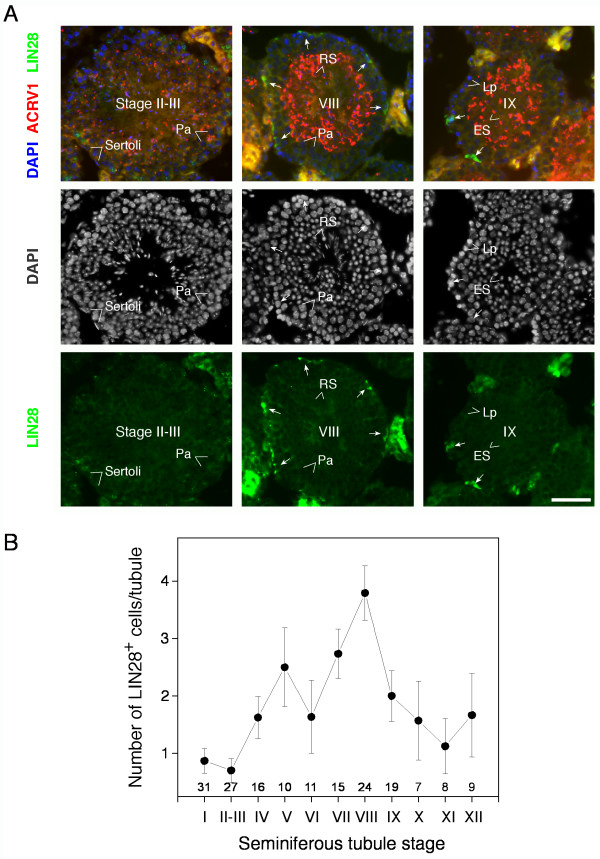
**Seminiferous epithelium stage-dependent distribution of LIN28-positive spermatogonia**. (**A**) Expression of LIN28 in representative tubules. Adult testis sections were immunostained with anti-LIN28 antibody (green) and anti-ACRV1 antibody (red). Chromatin was stained with DAPI (blue) but was presented in black and white in the second row of panels to show nuclear morphology and the amount of heterochromatin. The morphology of spermatid acrosomes and nuclei was used to determine the stages of seminiferous tubules and distinguish among various types of germ cells. The stage of each seminiferous tubule is shown as roman numerals in the center. LIN28-positive spermatogonia are indicated by arrows. Note that strong signal in interstitial cells (Leydig cells) is due to autofluorescence. Pa, pachytene spermatocyte; Lp, leptotene spermatocyte; RS, round spermatid; ES, elongating spermatid. Scale bar, 50 μm. (**B**) Frequency of LIN28-positive spermatogonia during spermatogenesis. A total of 177 seminiferous tubule cross-sections were examined for LIN28-positive spermatogonia. The count of tubule sections examined is shown above each stage (I-XII). The number of LIN28-positive cells per tubule section (mean ± SE) is plotted.

We next examined the expression of LIN28 by immunostaining of juvenile testis sections (Additional file 1). LIN28 was expressed in gonocytes from postnatal day 1-old mice and in spermatogonia from day 6- and day 14-old mice. LIN28 was predominantly cytoplasmic with punctate nuclear staining. Immunostaining analysis of adult testis sections revealed that LIN28-expressing cells were sparsely located at the periphery of seminiferous tubules (close to the basement membrane), suggesting that LIN28 is expressed in a subset of spermatogonia but not in meiotic and post-meiotic germ cells (Fig. 2A and Additional file 2). We also found that the number of LIN28-positive spermatogonia per tubule varied among the stages of seminiferous tubules (Fig. 2B). The seminiferous epithelium of mice is divided into twelve stages, each of which is defined by a unique association of differentiating germ cells [15]. For example, undifferentiated A_al _spermatogonia become differentiating A1 spermatogonia during stages VII-VIII [3]. Our results showed that the number of LIN28-positive spermatogonia peaked at stage VIII but decreased sharply at stage IX, indicating that LIN28 might be expressed in undifferentiated spermatogonia (Fig. 2B).

**Figure 3:**
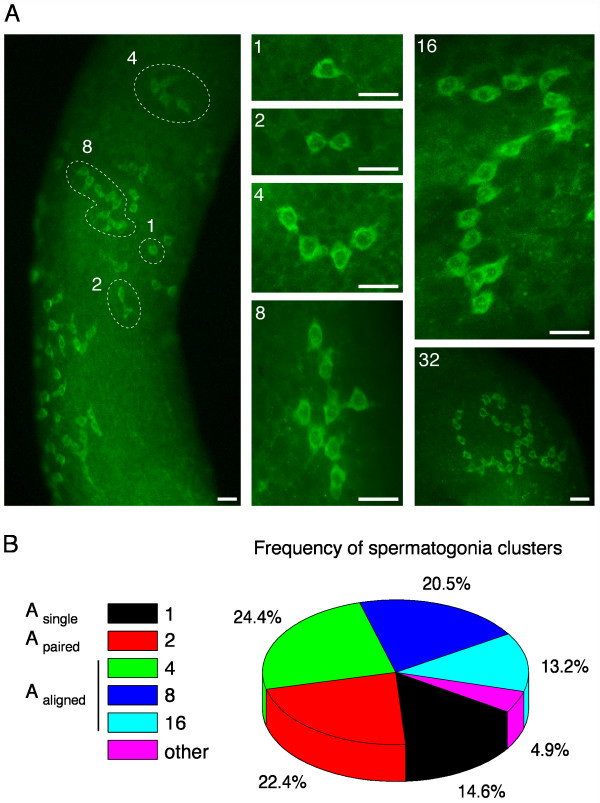
**LIN28 is specifically expressed in undifferentiated spermatogonia**. Whole-mount immunofluorescence of seminiferous tubules from adult mice was performed with anti-LIN28 antibody. (**A**) Whole-mount examination of LIN28 expression in seminiferous tubules. Examples of A_s_, A_pr_, and A_al _(up to 32 interconnected cells) spermatogonia are shown. LIN28 is predominantly cytoplasmic with punctate nuclear staining. Note that the A_pr _and A_al _spermatogonia are interconnected by intercellular cytoplasmic bridges due to incomplete cytokinesis. The background fluorescence helps orient the tubules. Scale bars, 25 μm. (**B**) Frequency of spermatogonia clusters comprising different numbers of chained LIN28-positive cells. A total of 205 isolated cells and clusters were counted. Only clearly identified clusters were included. The percentage of clusters with a longer chain of cells might be underestimated, since such large clusters extended around the tubule edge as shown in Fig. 3A and thus were excluded. Clusters with a non-2^n ^number of cells or too many chained cells were grouped as "other".

To address whether LIN28-expressing cells are indeed undifferentiated spermatogonia, we performed whole-mount immunofluorescent studies on dissected seminiferous tubules from adult testes. In these studies, undifferentiated spermatogonia can be definitively identified as A_s_, A_pr_, or A_al_. The A_pr _and A_al _spermatogonia are connected by intercellular cytoplasmic bridges as a chain of 2^n ^cells. We found that the expression of LIN28 was restricted to A_s _and the chained 2^n ^cells (1, 2, 4, 8, 16, or 32 cells) (Fig. 3A). A chain of 32-interconnected cells was very rare (Fig. 3). Chains with a non-2^n ^number of LIN28-positive cells were also observed at a low frequency (Fig. 3B). GFRA1 (GDNF receptor) is a marker of undifferentiated spermatogonia [16,17]. As expected, LIN28 was expressed in GFRA1-positive spermatogonia (Additional file 3). These whole-mount analyses demonstrated that LIN28 marks the undifferentiated spermatogonia.

### Expression of LIN28 in cultured spermatogonial stem cells (SSCs)

**Figure 4:**
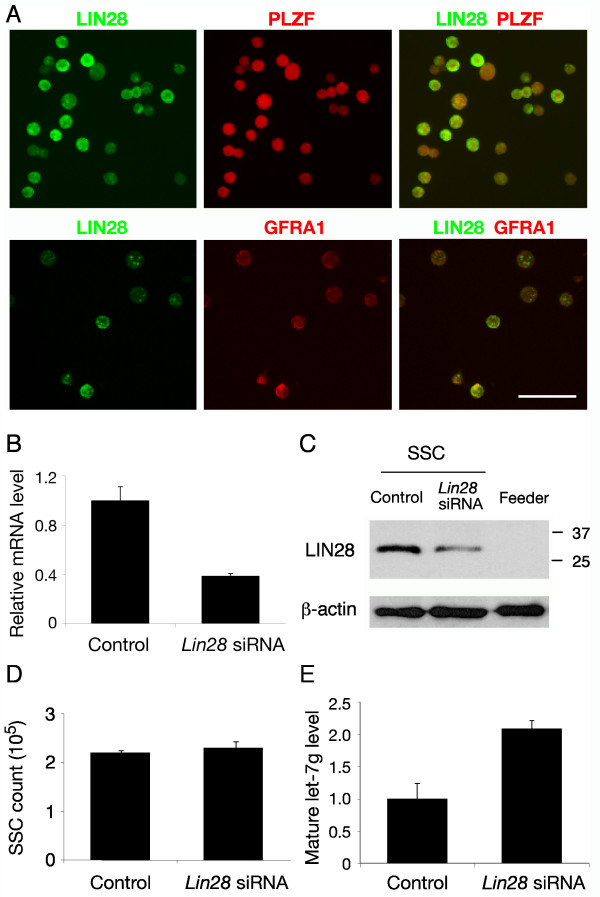
**Expression and siRNA knockdown of LIN28 in cultured spermatogonia highly enriched for spermatogonial stem cells (SSCs)**. (**A**) Immunostaining of SSCs with anti-LIN28 and anti-PLZF or anti-GFRA1 antibodies. Scale bar, 50 μm. (**B**) Quantitative PCR measurement of *Lin28 *mRNA levels (n = 3, mean ± SE) in SSCs after siRNA treatment for 30 hours. (**C**) Decreased LIN28 protein abundance (43% compared to the control) in SSCs after 30 hours of siRNA treatment. The control SSCs were not treated with Lin28 siRNA. Feeder cells served as a negative control. β-actin served as a loading control. (**D**) The number of SSCs (n = 3, mean ± SE) with and without *Lin28 *siRNA treatment. (**E**) Quantitative measurement of mature let-7g miRNA levels (n = 3, mean ± SE) in SSCs after siRNA treatment for 30 hours.

Spermatogonial stem cells (SSCs) are believed to be a subset of A_s _cells [3]. Currently, there are no cytological markers that could distinguish SSCs from "non-stem" A_s _cells. To examine whether LIN28 is expressed in SSCs, we performed double immunostaining of cultured spermatogonia highly enriched for SSCs with anti-LIN28 and anti-PLZF or anti-GFRA1 antibodies. PLZF is required for maintenance of SSCs [18,19]. We found that LIN28 was expressed in cultured SSCs, but the abundance of LIN28 in SSCs was not uniform, suggesting the heterogeneity of in vitro cultured SSCs (Fig. 4A and Additional file 2).

In an attempt to determine the role of *Lin28 *in the maintenance of SSCs, we treated SSCs with *Lin28 *siRNAs. The siRNA knockdown decreased the level of *Lin28 *mRNA by 60% and consequently reduced the abundance of LIN28 protein by nearly 60% (Fig. 4B, C). However, siRNA treatment did not causes a change in the total number of cultured cells (Fig. 4D), suggesting that the remaining LIN28 protein might be sufficient for maintaining SSC or that LIN28 is dispensable for the survival of SSCs.

Several recent studies have demonstrated that LIN28 is a negative regulator of let-7 microRNA biogenesis in embryonic stem cells and other stem cells [20-24]. Specifically, LIN28 prevents Dicer from processing let-7 microRNAs by mediating the terminal uridylation of let-7 microRNA precursors [21]. In agreement with these studies, siRNA knockdown of LIN28 in cultured SSCs led to an increased level of mature let-7g miRNA (Fig. 4E).

### *Ngn3*-GFP labels a more committed subpopulation of LIN28-positive spermatogonia

**Figure 5:**
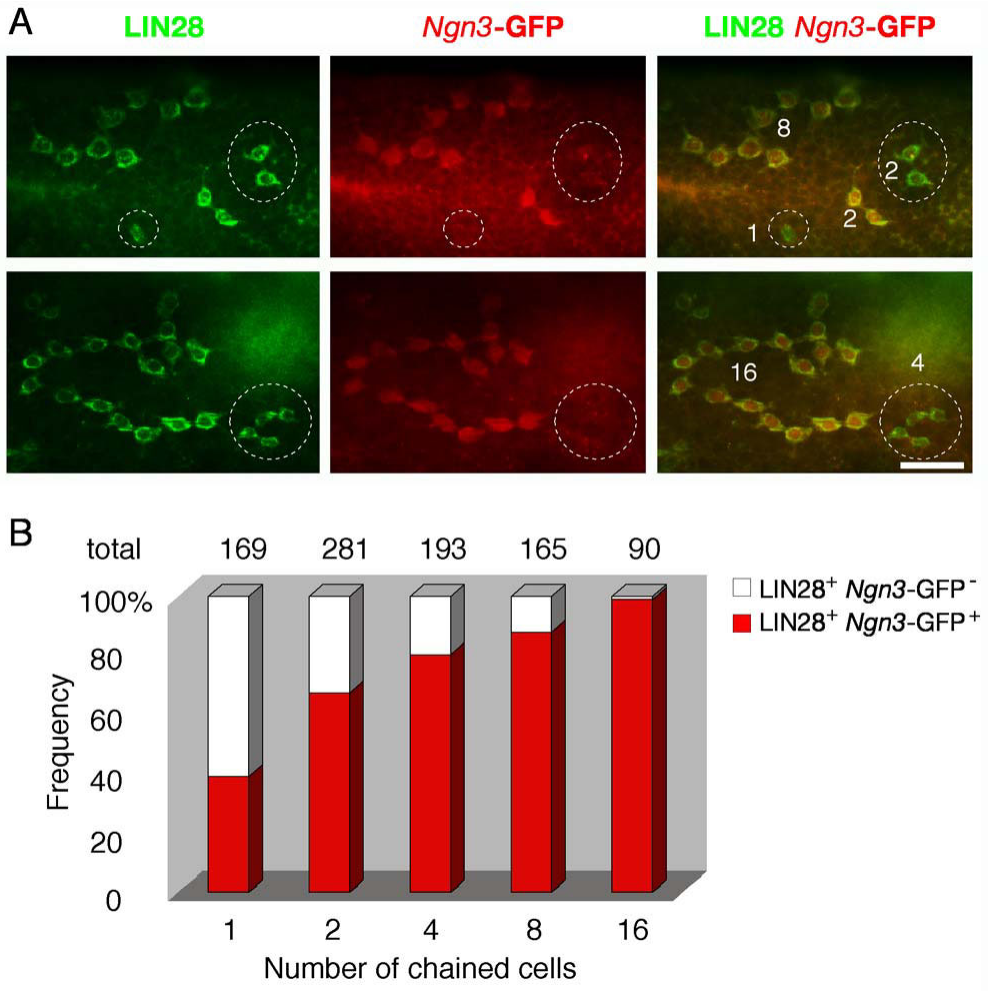
***Ngn3*-GFP labels a more committed subpopulation of LIN28-positive spermatogonia. **Seminiferous tubules from adult *Ngn3*-GFP mice were immunostained with anti-LIN28 and anti-GFP antibodies [26]. We used antibodies to visualize GFP, since the GFP fluorescence was weak. (**A**) LIN28-positive undifferentiated spermatogonia are divided into *Ngn3*-GFP-positive and *Ngn3*-GFP-negative subpopulations. A_s_, Apr and the number of A_al _spermatogonia were indicated. *Ngn3*-GFP-negative spermatogonia were circled. Scale bar, 25 μm. (**B**) Frequency of spermatogonia clusters (2^n ^cells: 1, 2, 4, 8, 16) with cells that are either all *Ngn3*-GFP-positive or all *Ngn3*-GFP-negative. The total number of 2^n^-cell clusters examined was shown above each column.

**Figure 6:**
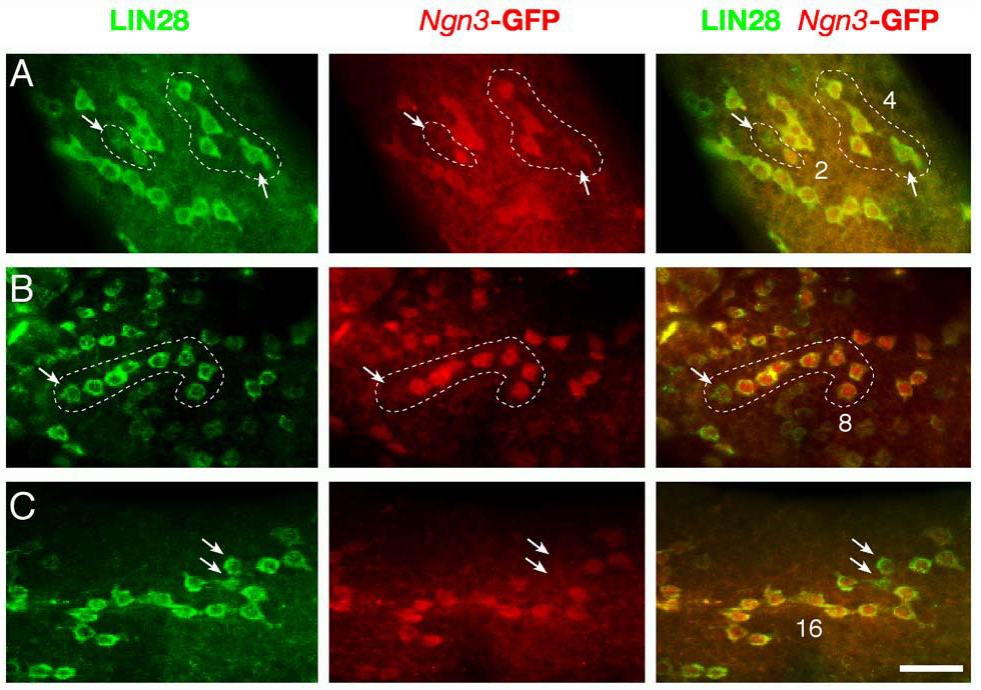
**Heterogeneity of *Ngn3*-GFP-expression in A_pr _and A_al _spermatogonia**. Seminiferous tubules from adult *Ngn3*-GFP mice were immunostained with anti-LIN28 and anti-GFP antibodies. (**A**) Presence of *Ngn3*-GFP-negative cell(s) in A_pr _and A_al _(4-cell) spermatogonia. Note the unusual 4-cell chain (encircled) that is branched. (**B**) One cell (arrow) at the end of the 8-cell chain was *Ngn3*-GFP-negative. (**C**) Two spermatogonia (arrows) in the middle of 16-cell chain were *Ngn3*-GFP-negative. Scale bar, 25 μm.

*Ngn3 *is specifically expressed in undifferentiated spermatogonia (A_s _to A_al_) [25]. To determine if *Ngn3 *and *Lin28 *mark the same population of undifferentiated spermatogonia, we made use of *Ngn3*-GFP mice, in which GFP was inserted into the *Ngn3 *locus by gene replacement [26]. We performed whole-mount immunostaining of *Ngn3*-GFP seminiferous tubules with anti-LIN28 and anti-GFP antibodies. This analysis revealed that only a subpopulation of LIN28-positive spermatogonia was GFP-positive (Fig. 5A). Overall, ~40% of LIN28-positive A_s _spermatogonia were GFP-positive, supporting that the population of A_s _cells were not homogeneous. The A_pr _and A_al _spermatogonia were either all GFP-positive or all GFP-negative (Fig. 5A) except a few as described later (Fig. 6). Interestingly, the percentage of *Ngn3*-GFP-positive spermatogonia increased dramatically as spermatogonia develop from A_s _to A_al _(16 cells) (Fig. 5B). While ~40% of A_s _cells were GFP-positive, nearly all A_al _(16-cell) spermatogonia were GFP-positive. As the number of chained cells increases, spermatogonia become more and more committed to differentiation. Taken together, our data suggested that *Ngn3 *delineates a more committed subpopulation of undifferentiated spermatogonia, in contrast, the LIN28-positive but *Ngn3*-GFP-negative spermatogonia are more primitive.

We observed heterogeneity of *Ngn3*-GFP expression among A_pr _and A_al _spermatogonia (Fig. 6). In A_pr _spermatogonia, one cell was GFP-positive and the other was GFP-negative (Fig. 6A). In an 8-cell chain of A_al _spermatogonia, seven cells were GFP-positive but one was GFP-negative (Fig. 6B). In a 16-cell chain of A_al _spermatogonia, two cells in the middle of the chain were GFP-negative (Fig. 6C). Twelve out of 710 clusters examined (1.7%) were found to contain both GFP-positive and GFP-negative cells in the same chain (one A_pr_, two 4-cell A_al_, six 8-cell A_al_, and three 16-cell A_al _spermatogonia). The presence of *Ngn3*-GFP-negative cells in a chain of GFP-positive spermatogonia suggested that the GFP-negative cells might have de-differentiated and thus reverted to a more primitive (stem cell) fate.

## Discussion

The transition from undifferentiated A_al _to differentiating A1 spermatogonia is a critical point during spermatogonial development and is tightly regulated [3,5,27]. This transition is specifically perturbed by several conditions, including cryptorchidism, Vitamin A deficiency, and *Steel *and *c-kit *mutations [28-31]. In this study, we found that LIN28, a pluripotency factor, is specifically expressed in the undifferentiated (A_s _to A_al_) spermatogonia, suggesting that it might play a role in maintaining the undifferentiated state in spermatogonia. *Lin28 *is expressed in mouse and human embryonic stem cells, embryonic carcinoma cells, neural stem cells, and diverse embryonic tissues [10,13,24,32]. Recently, LIN28, together with OCT4, SOX2, and NANOG, was used to reprogram human fibroblasts to pluripotent stem cells [12]. In mammalian cultured cells, the expression of LIN28 appears to be associated with "stemness" [33]. Very recent studies have discovered a feedback loop, in which LIN28 blocks the maturation of the let-7 microRNAs and *Lin28 *is downregulated by let-7 [20,24]. Specifically, LIN28 prevents the processing of let-7 precursor microRNAs by Dicer through mediating the terminal uridylation of let-7 precursors [21]. Notably, LIN28 is not essential for reprogramming human fibroblasts into pluripotent stem cells but does increase the reprogramming efficiency [12]. The siRNA knockdown experiments suggested that LIN28 might not be essential for self-renewal of human ES cells [32]. We tested the role of LIN28 in the maintenance of SSCs by siRNA knockdown. The siRNA treatment did not cause a change in the total number of cells in culture, suggesting that LIN28 might be dispensable for maintenance of SSC or that the remaining LIN28 protein after knockdown might be sufficient for its full function. However, consistent with the known function of LIN28 in blockade of let-7 miRNA processing [20,24], we found that siRNA knockdown of LIN28 in cultured SSCs caused an increased level of mature let-7g miRNA. In a recent study of five genes (*Bcl6b*, *Etv5*, *Bhlhe40*, *Hoxc4*, and *Tec*) involved in the SSC self renewal, siRNA treatment caused a decrease in the number of SSC stem cells as determined by transplantation without changing the total number of cells in culture [34]. Therefore, the possible involvement of LIN28 in SSC self-renewal remains to be determined by siRNA treatment followed by transplantation in future studies.

*Ngn3 *is also specifically expressed in undifferentiated spermatogonia in mouse [25]. Pulse-chase labeling studies using *Ngn3*/Cre™ CAG-CAT-Z transgenic (driven by 6.7 kb *Ngn3 *upstream sequence) mice identified two compartments of spermatogonial stem cells: the actual stem cells and the potential stem cells [35]. In a normal situation, the actual stem cells undergo self-renewal and give rise to transit cells that further divide to become terminally differentiated cells. The transit cells, immediate progeny of actual stem cells, are potential stem cells, in a sense that they can function as stem cells in the case of loss of actual stem cells or when transplanted [35,36]. Nakagawa et al showed that *Ngn3*-Cre-mediated pulse-labeled spermatogonia contributed to only 0.3% of actual stem cells and to 11.7% of potential stem cells. However, it is difficult to image that such low percentages of contribution to stem cells might be entirely due to the low efficiency of *Ngn3*/Cre-mediated recombination as previously discussed [35].

We have demonstrated that the population of undifferentiated spermatogonia is cytologically divided into two subpopulations: *Ngn3*-GFP-negative and *Ngn3*-GFP-positive. A_s _cells, the most primitive type of undifferentiated spermatogonia, are heterogeneous. More than 40% of LIN28-positive A_s _spermatogonia are *Ngn3*-GFP-negative. The percentage of *Ngn3*-GFP-positive clusters increases progressively with the chain length of interconnected undifferentiated spermatogonia (2-, 4-, 8-, 16-cell clusters), suggesting that *Ngn3*-GFP-expressing spermatogonia are more committed to differentiation (with low stem cell activity), while *Ngn3*-GFP-negative ones are more primitive (with high stem cell activity). We hypothesize that the low contribution of *Ngn3*-Cre-mediated pulse-labeled cells to stem cells found in the previous study [35] is more likely attributed to the previously unknown population of *Ngn3*-negative undifferentiated spermatogonia. Therefore, our current studies together with the pulse-chase labeling experiments done by Nakagawa et al [35] show that the *Ngn3*-positive cells contain few (0.3%) actual stem cells and some potential stem cells (11.7%). By inference, these studies suggest that the *Ngn3*-negative undifferentiated spermatogonia might contain >99% of the actual stem cells and nearly 90% of potential stem cells.

According to the A_s _model, A_s _(single) spermatogonia and a few A_pr _(false pairs) can act as stem cells [1-3]. In this model, the A_s _spermatogonium divides either to produce two new stem cells if separate or to become A_pr _if two daughter cells remain connected by an intercellular bridge. However, it remains unknown whether A_pr _and A_al _spermatogonia in mouse could potentially act as stem cells. In *Drosophila *testis and ovary, transit-amplifying germ cells can dedifferentiate and revert into functional stem cells [37,38]. Recently, c-kit-positive (differentiating) spermatogonia were shown to be able to revert to functional stem cells when transplanted into testis [39]. Studies of CDH1-expressing spermatogonia showed heterogeneous expression of *c-Kit *and *Tacstd1 *among undifferentiated spermatogonia, lending support for de-differentiation in mouse [40]. In the current study of mouse testis, we have observed that, in the same chain of A_al _spermatogonia, one or two cells are *Ngn3*-GFP-negative, while the remaining cells are *Ngn3*-GFP-positive, suggesting that *Ngn3*-GFP-negative cells in the A_al _spermatogonia might have reverted to a more primitive state.

## Conclusion

In this study, we have shown that LIN28, a pluripotency factor, is specifically expressed in undifferentiated spermatogonia in mice, suggesting that it might play a role in maintenance of the undifferentiated state of this primitive germ cell population. We have also found that the undifferentiated spermatogonia exist as two subpopulations: *Ngn3*-GFP-negative (high stem cell potential) and *Ngn3*-GFP-positive (high differentiation commitment). In addition, our study provides cytological evidence supporting dedifferentiation of spermatogonia in mice.

## Methods

### Western blot analysis

Mouse tissues were homogenized using a glass homogenizer in the extraction buffer (62.5 mM Tris-HCl, pH 6.8, 3% SDS, 10% glycerol, 5% β-mercaptoethanol). Protein lysate (20 μg) was separated on 12% SDS-PAGE gels and electro-blotted onto PVDF membranes. Western blotting was performed using the following antibodies: goat anti-LIN28 antibody (1:100, Cat# AF3757, R&D Systems) and anti-β-actin monoclonal antibody (1:2,500, Cat# A5441, Sigma-Aldrich). HRP-conjugated secondary antibodies were used (Sigma-Aldrich).

### Immunofluorescence microscopy

To prepare frozen sections, testes from C57BL/6J mice of postnatal day 1, 6, 14 or 2-month (adult) were fixed in 4% paraformaldehyde (PFA) at 4°C for 8 hours and were dehydrated in 30% (w/v) sucrose overnight. Testes were embedded with Neg 50 tissue freezing solution (Cat# 6502, Thermo Scientific) and frozen in dry ice/ethanol. Sections (8 μm) were cut using a Reichert-Jung cryo-microtome and then post-fixed in 4% PFA at room temperature for 10 minutes prior to immunostaining.

For whole-mount analysis, seminiferous tubules from adult (2-month-old) C57BL/6J mice were prepared as previously described with modifications [41]. Briefly, testis tubules were washed once with PBS, fixed in 5 ml 4% PFA for 3 hours, and incubated sequentially with 5 ml of 25%, 50%, 75% and 100% TBST (1×TBS containing 0.1% Tween 20) at 4°C each for 30 minutes. Testis tubules were frozen in 1×TBS at -20°C. Immunostaining of testis sections, whole mounts of seminiferous tubules, or SSCs was performed with the following primary antibodies: goat anti-LIN28 (1:100), guinea pig anti-ACRV1 (1:500, gift from PP Reddi) [42], rabbit anti-GFP (1:500, Cat# Ab6556, Abcam), anti-PLZF (1:200, Cat# OP128L, Calbiochem), and anti-GFRA1 (1:20, Cat# sc-10716, Santa Cruz Biotech). Texas red or FITC-conjugated secondary antibodies were used (Vector Laboratories). Nuclear DNA was stained with DAPI provided in mounting medium. Samples were visualized under a Zeiss Axioskop 40 fluorescence microscope. Images were captured with an Evolution QEi digital camera (MediaCybernetics) and processed with the Image-Pro software (Phase 3 Imaging Systems).

### *Ngn3*-GFP and XX^Y* ^mice

The derivation of *Ngn3*-GFP mice has been described previously [26]. In *Ngn3*-GFP mice, the enhanced green fluorescent protein (eGFP) substitutes the *Ngn3 *coding region through gene replacement; thus GFP is under the transcriptional control of all endogenous *Ngn3 *regulatory elements. Adult (2-month-old) *Ngn3*-GFP heterozygous mice on a mixed (129/C57BL/6) genetic background were used, because homozygous (*Ngn3*^-/-^) mice die by postnatal day 3. XX^Y*^ mice were generated by breeding XY* males with wild type females [14]. The care and use of mice were within standard ethical guidelines and were approved by the Institutional Animal Care and Use Committee at the University of Pennsylvania.

### SSC enrichment, SSC culture, siRNA transfection, and qPCR analysis

Mouse spermatogonia highly enriched for spermatogonial stem cells (SSCs) were prepared and cultured as previously described [43,44]. Briefly, single-cell suspensions were prepared from eight testes from post-natal day 6~8 C57BL/6 pups by digestion with Trypsin-EDTA (0.25%, Invitrogen) and DNase I (7 mg/ml, Sigma). Cell suspensions were layered on top of a 30% Percoll solution and were centrifuged to enrich germ cells. After resuspension, SSCs were isolated by magnetic activated cell sorting (MACS) using Thy1.2 antibody-conjugated microbeads (Cat#130-049-101, Miltenyi Biotec). Thy1^+ ^cells were seeded at a density of 0.5 – 1.0 × 10^5 ^cells per well on 12-well culture plates with mitomycin C-treated STO feeders. Self-renewing SSCs were cultured in a chemically defined serum-free MEMα medium (Invitrogen) containing 0.2% BSA, 10 μg/ml Transferrin, 7.6 μeq/L free fatty acids, 3 × 10^-8 ^M Na_2_SeO_3_, 50 μM β-ME, 5 μg/ml Insulin, 60 μM Putrescine, 2 mM L-glutamine, and 10 mM HEPES), 20 ng/ml GDNF (R&D Systems), 150 ng/ml soluble GFRα1 (R&D Systems), and 1 ng/ml bFGF (BD Biosciences). The medium was changed every 2–3 days. All cultures were maintained at 37°C in a humidified 5% CO_2 _incubator. Cells were passaged at 7-day intervals at 1:2–3 dilution.

Mouse *Lin28 *siRNAs (On-target plus Smartpool, Cat# L-050153, Thermo Scientific Dharmacon) was used. Silence^® ^siRNA served as a negative control (Cat# AM4611, Ambion). After trypsin digestion and washing, SSCs were plated into wells of a 12-well dish without feeders in the antibiotic-free culture medium at a density of 2 × 10^5 ^cells/well. Cells were allowed to settle for 2–3 hours prior to siRNA treatment. For each well, 75 pmol of siRNA and 2 μl of LipofectamineTM RNAiMAX reagent (Invitrogen) were mixed with 200 μl of OptiMEM (Invitrogen). After the 30-hour incubation, total RNA and proteins were prepared for qPCR and western blotting. Quantitative RT-PCR (qPCR) analysis was performed using SYBR green on an ABI 7300 sequence detection system with the following *Lin28 *primers: AGACCAACCATTTGGAGTGC and AATCGAAACCCGTGAGACAC. Level of mature let-7g miRNA was measured by using specific TaqMan probes per the manufacturer's instructions (Applied Biosystems). Quantification of *Lin28 *and mature let-7g transcript levels was normalized to *Rps2 *(ribosomal protein S2) within the log phase of the amplification curve. For SSC count, 1 × 10^5 ^cells/well after 30-hour siRNA treatment were plated onto fresh feeders, cultured in a defined serum-free media with 20 ng/ml GDNF, 150 ng/ml GFRα1 and 1 ng/ml bFGF for 7 days. Each experiment was performed on three independent SSC lines.

## Authors' contributions

KZ and XW performed experiments. XW and KHK contributed material. PJW and KZ wrote the manuscript. All authors have seen, commented, and approved the final manuscript.

## Supplementary Material

Additional file 1 **Localization of LIN28 in juvenile testes**. Frozen sections of mouse postnatal testis (day 1, 6, and 14) were immunostained with anti-LIN28 antibodies (green) and DAPI (blue). Arrows indicate LIN28-positive spermatogonia in seminiferous tubules. Note that LIN28-speramtogonia contain no or little heterochromatin, characteristic of undifferentiated spermatogonia. Scale bar, 50 μm. Click here for file

Additional file 2 **Negative controls for immunostaining with anti-LIN28 antibody**. (A) Adjacent frozen sections of adult mouse testis were immunostained with (right panel) or without (left panel) anti-LIN28 antibodies (green). In the control section (left), the primary antibody (anti-LIN28) was omitted. Nuclear DNA was stained with DAPI (blue). Composite images from three channels (red, green, blue) were presented to show the autofluorescence of interstitial cells such as Leydig cells indicated by arrowheads. Arrows indicate LIN28-positive spermatogonia in seminiferous tubules. (B) Immunostaining of cultured SSCs with anti-LIN28 and anti-PLZF antibodies (right panel). In the control (left) panel, both primary antibodies were omitted, and only low level of background signal was observed. Scale bar, 50 μm. Click here for file

Additional file 3 **Expression of LIN28 in GFRA1-positive spermatogonia**. Seminiferous tubules from adult mice were immunostained with anti-LIN28 and anti-GFRA1 antibodies. A_s_, A_pr_, and A_al _spermatogonia were encircled. Scale bar, 25 μm. Click here for file

## References

[B1] Huckins C (1971). The spermatogonial stem cell population in adult rats. I. Their morphology, proliferation and maturation. Anat Rec.

[B2] Oakberg EF (1971). Spermatogonial stem-cell renewal in the mouse. Anat Rec.

[B3] de Rooij DG (1998). Stem cells in the testis. Int J Exp Pathol.

[B4] Brinster RL (2007). Male germline stem cells: from mice to men. Science.

[B5] de Rooij DG, Russell LD (2000). All you wanted to know about spermatogonia but were afraid to ask. J Androl.

[B6] Shinohara T, Orwig KE, Avarbock MR, Brinster RL (2000). Spermatogonial stem cell enrichment by multiparameter selection of mouse testis cells. Proc Natl Acad Sci USA.

[B7] Wang PJ, McCarrey JR, Yang F, Page DC (2001). An abundance of X-linked genes expressed in spermatogonia. Nat Genet.

[B8] Wang PJ, Page DC, McCarrey JR (2005). Differential expression of sex-linked and autosomal germ-cell-specific genes during spermatogenesis in the mouse. Hum Mol Genet.

[B9] Moss EG, Lee RC, Ambros V (1997). The cold shock domain protein LIN-28 controls developmental timing in C. elegans and is regulated by the lin-4 RNA. Cell.

[B10] Moss EG, Tang L (2003). Conservation of the heterochronic regulator Lin-28, its developmental expression and microRNA complementary sites. Dev Biol.

[B11] Seggerson K, Tang L, Moss EG (2002). Two genetic circuits repress the Caenorhabditis elegans heterochronic gene lin-28 after translation initiation. Dev Biol.

[B12] Yu J, Vodyanik MA, Smuga-Otto K, Antosiewicz-Bourget J, Frane JL, Tian S, Nie J, Jonsdottir GA, Ruotti V, Stewart R, Slukvin II, Thomson JA (2007). Induced pluripotent stem cell lines derived from human somatic cells. Science.

[B13] Yang DH, Moss EG (2003). Temporally regulated expression of Lin-28 in diverse tissues of the developing mouse. Gene Expr Patterns.

[B14] Hunt PA, Eicher EM (1991). Fertile male mice with three sex chromosomes: evidence that infertility in XYY male mice is an effect of two Y chromosomes. Chromosoma.

[B15] Russell LD, Ettlin RA, Sinha Hikim AP, Clegg ED (1990). Histological and Histopathological Evaluation of the Testis.

[B16] Buageaw A, Sukhwani M, Ben-Yehudah A, Ehmcke J, Rawe VY, Pholpramool C, Orwig KE, Schlatt S (2005). GDNF family receptor alpha1 phenotype of spermatogonial stem cells in immature mouse testes. Biol Reprod.

[B17] Hofmann MC, Braydich-Stolle L, Dym M (2005). Isolation of male germ-line stem cells; influence of GDNF. Dev Biol.

[B18] Buaas FW, Kirsh AL, Sharma M, McLean DJ, Morris JL, Griswold MD, de Rooij DG, Braun RE (2004). Plzf is required in adult male germ cells for stem cell self-renewal. Nat Genet.

[B19] Costoya JA, Hobbs RM, Barna M, Cattoretti G, Manova K, Sukhwani M, Orwig KE, Wolgemuth DJ, Pandolfi PP (2004). Essential role of Plzf in maintenance of spermatogonial stem cells. Nat Genet.

[B20] Viswanathan SR, Daley GQ, Gregory RI (2008). Selective blockade of microRNA processing by Lin28. Science.

[B21] Heo I, Joo C, Cho J, Ha M, Han J, Kim VN (2008). Lin28 mediates the terminal uridylation of let-7 precursor MicroRNA. Mol Cell.

[B22] Piskounova E, Viswanathan SR, Janas M, LaPierre RJ, Daley GQ, Sliz P, Gregory RI (2008). Determinants of microRNA processing inhibition by the developmentally regulated RNA-binding protein Lin28. J Biol Chem.

[B23] Newman MA, Thomson JM, Hammond SM (2008). Lin-28 interaction with the Let-7 precursor loop mediates regulated microRNA processing. RNA.

[B24] Rybak A, Fuchs H, Smirnova L, Brandt C, Pohl EE, Nitsch R, Wulczyn FG (2008). A feedback loop comprising lin-28 and let-7 controls pre-let-7 maturation during neural stem-cell commitment. Nat Cell Biol.

[B25] Yoshida S, Takakura A, Ohbo K, Abe K, Wakabayashi J, Yamamoto M, Suda T, Nabeshima Y (2004). Neurogenin3 delineates the earliest stages of spermatogenesis in the mouse testis. Dev Biol.

[B26] Lee CS, Perreault N, Brestelli JE, Kaestner KH (2002). Neurogenin 3 is essential for the proper specification of gastric enteroendocrine cells and the maintenance of gastric epithelial cell identity. Genes Dev.

[B27] de Rooij DG, de Boer P (2003). Specific arrests of spermatogenesis in genetically modified and mutant mice. Cytogenet Genome Res.

[B28] Nishimune Y, Haneji T (1981). Testicular DNA synthesis in vivo: comparison between unilaterally cryptorchid testis and contralateral intact testis in mouse. Arch Androl.

[B29] van Pelt AM, van Dissel-Emiliani FM, Gaemers IC, Burg MJ van der, Tanke HJ, de Rooij DG (1995). Characteristics of A spermatogonia and preleptotene spermatocytes in the vitamin A-deficient rat testis. Biol Reprod.

[B30] Koshimizu U, Sawada K, Tajima Y, Watanabe D, Nishimune Y (1991). White-spotting mutations affect the regenerative differentiation of testicular germ cells: demonstration by experimental cryptorchidism and its surgical reversal. Biol Reprod.

[B31] Tajima Y, Sakamaki K, Watanabe D, Koshimizu U, Matsuzawa T, Nishimune Y (1991). Steel-Dickie (Sld) mutation affects both maintenance and differentiation of testicular germ cells in mice. J Reprod Fertil.

[B32] Darr H, Benvenisty N (2009). Genetic analysis of the role of the reprogramming gene LIN-28 in human embryonic stem cells. Stem Cells.

[B33] Richards M, Tan SP, Tan JH, Chan WK, Bongso A (2004). The transcriptome profile of human embryonic stem cells as defined by SAGE. Stem Cells.

[B34] Schmidt JA, Avarbock MR, Tobias JW, Brinster RL (2009). Identification of GDNF-Regulated Genes Important for Spermatogonial Stem Cell Self-Renewal in the Rat. Biol Reprod.

[B35] Nakagawa T, Nabeshima Y, Yoshida S (2007). Functional identification of the actual and potential stem cell compartments in mouse spermatogenesis. Dev Cell.

[B36] Potten CS, Loeffler M (1990). Stem cells: attributes, cycles, spirals, pitfalls and uncertainties. Lessons for and from the crypt. Development.

[B37] Brawley C, Matunis E (2004). Regeneration of male germline stem cells by spermatogonial dedifferentiation in vivo. Science.

[B38] Kai T, Spradling A (2004). Differentiating germ cells can revert into functional stem cells in Drosophila melanogaster ovaries. Nature.

[B39] Barroca V, Lassalle B, Coureuil M, Louis JP, Le Page F, Testart J, Allemand I, Riou L, Fouchet P (2009). Mouse differentiating spermatogonia can generate germinal stem cells in vivo. Nat Cell Biol.

[B40] Tokuda M, Kadokawa Y, Kurahashi H, Marunouchi T (2007). CDH1 is a specific marker for undifferentiated spermatogonia in mouse testes. Biol Reprod.

[B41] Greenbaum MP, Yan W, Wu MH, Lin YN, Agno JE, Sharma M, Braun RE, Rajkovic A, Matzuk MM (2006). TEX14 is essential for intercellular bridges and fertility in male mice. Proc Natl Acad Sci USA.

[B42] Reddi PP, Naaby-Hansen S, Aguolnik I, Tsai JY, Silver LM, Flickinger CJ, Herr JC (1995). Complementary deoxyribonucleic acid cloning and characterization of mSP-10: the mouse homologue of human acrosomal protein SP-10. Biol Reprod.

[B43] Kubota H, Avarbock MR, Brinster RL (2004). Culture conditions and single growth factors affect fate determination of mouse spermatogonial stem cells. Biol Reprod.

[B44] Oatley JM, Brinster RL (2006). Spermatogonial stem cells. Methods Enzymol.

