# Supplementary figures and images for: The pluripotency factor LIN28 marks undifferentiated spermatogonia in mouse
Source: BMC Dev Biol. 2009 Jun 29;9:38. doi: 10.1186/1471-213X-9-38 (PMC2719617; doi:10.1186/1471-213X-9-38)

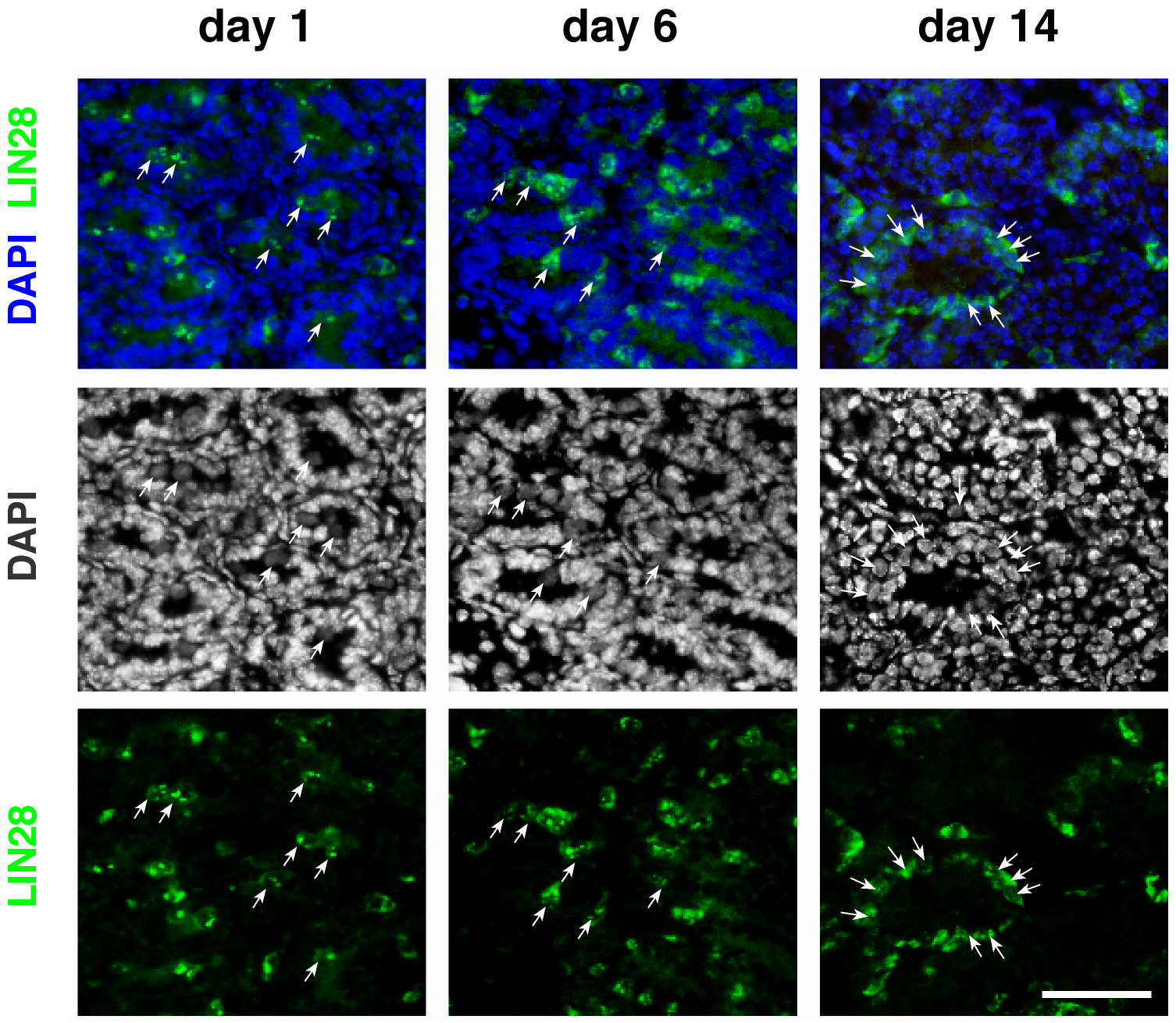

Supplement: Additional file 1 — Localization of LIN28 in juvenile testes. Frozen sections of mouse postnatal testis (day 1, 6, and 14) were immunostained with anti-LIN28 antibodies (green) and DAPI (blue). Arrows indicate LIN28-positive spermatogonia in seminiferous tubules. Note that LIN28-speramtogonia contain no or little heterochromatin, characteristic of undifferentiated spermatogonia. Scale bar, 50 μm. [file 1471-213X-9-38-S1.tiff]

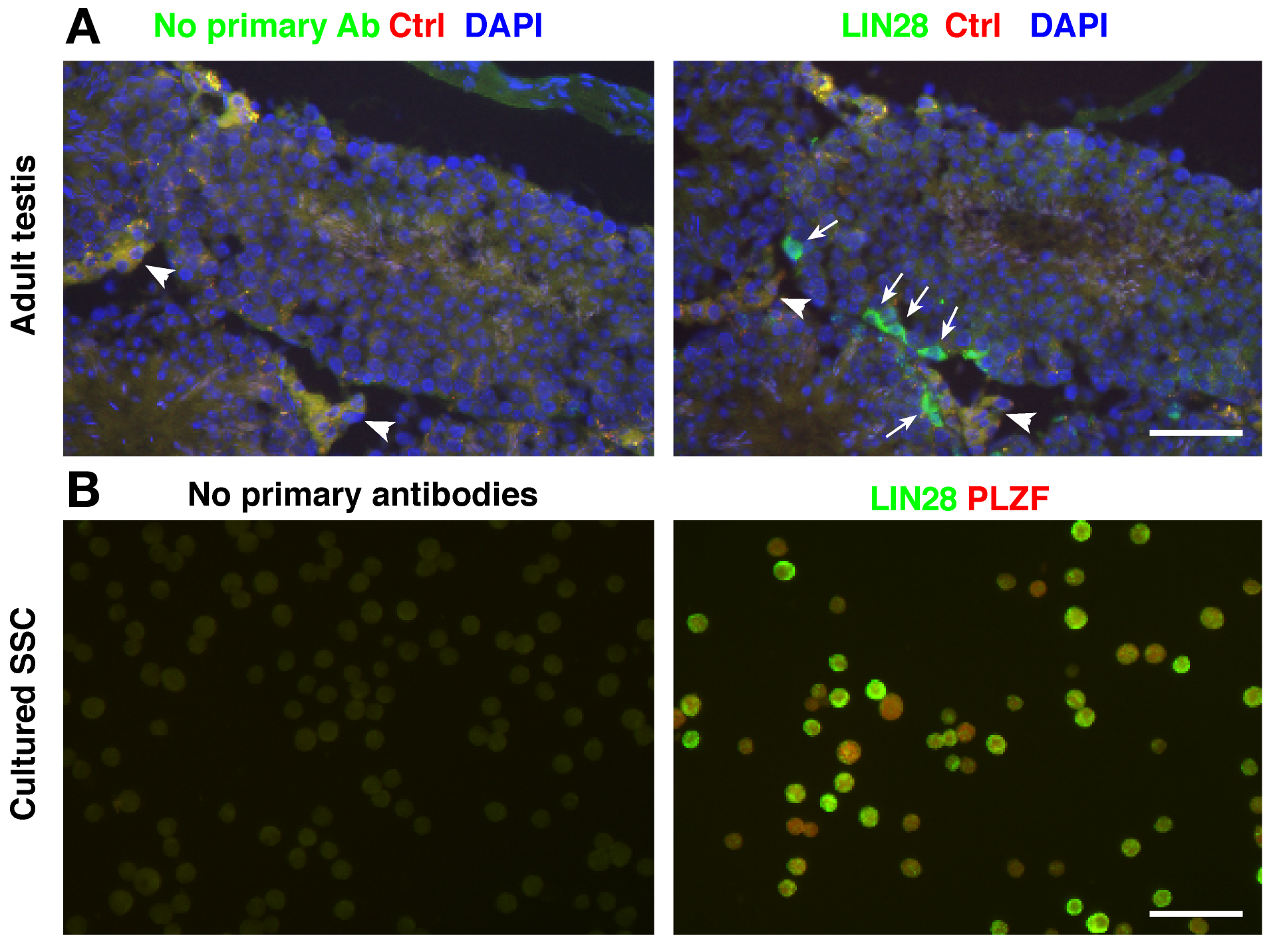

Supplement: Additional file 2 — Negative controls for immunostaining with anti-LIN28 antibody. (A) Adjacent frozen sections of adult mouse testis were immunostained with (right panel) or without (left panel) anti-LIN28 antibodies (green). In the control section (left), the primary antibody (anti-LIN28) was omitted. Nuclear DNA was stained with DAPI (blue). Composite images from three channels (red, green, blue) were presented to show the autofluorescence of interstitial cells such as Leydig cells indicated by arrowheads. Arrows indicate LIN28-positive spermatogonia in seminiferous tubules. (B) Immunostaining of cultured SSCs with anti-LIN28 and anti-PLZF antibodies (right panel). In the control (left) panel, both primary antibodies were omitted, and only low level of background signal was observed. Scale bar, 50 μm. [file 1471-213X-9-38-S2.tiff]

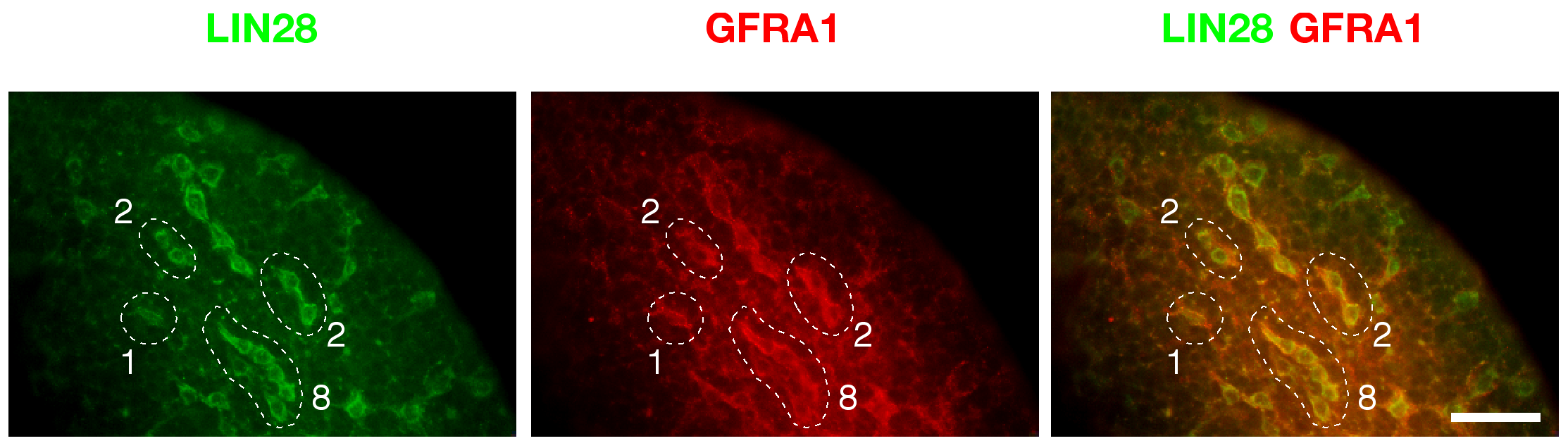

Supplement: Additional file 3 — Expression of LIN28 in GFRA1-positive spermatogonia. Seminiferous tubules from adult mice were immunostained with anti-LIN28 and anti-GFRA1 antibodies. As, Apr, and Aal spermatogonia were encircled. Scale bar, 25 μm. [file 1471-213X-9-38-S3.tiff]
